# Do the SRS-22 self-image and mental health domain scores reflect the degree of asymmetry of the back in adolescent idiopathic scoliosis?

**DOI:** 10.1186/s13013-017-0144-9

**Published:** 2017-12-11

**Authors:** James Cheshire, Adrian Gardner, Fiona Berryman, Paul Pynsent

**Affiliations:** 1 Institute of Metabolism and Systems Research (IMSR), University of Birmingham, Birmingham, UK; 2 The Royal Orthopaedic Hospital NHS Foundation Trust, Birmingham, UK; 3 Department of Anatomy, Institute of Clinical Science, University of Birmingham, Birmingham, UK

**Keywords:** Adolescent idiopathic scoliosis (AIS), Surface topography, Scoliosis Research Society-22 (SRS-22), Patient-reported outcomes, Health-related quality of life (HRQOL), ISIS2

## Abstract

**Background:**

Patient-reported outcomes are becoming increasingly recognised in the management of patients with adolescent idiopathic scoliosis (AIS). Integrated Shape Imaging System 2 (ISIS2) surface topography is a validated tool to assess AIS. Previous studies have failed to demonstrate strong correlations between AIS and patient-reported outcomes highlighting the need for additional objective surface parameters to define the deformities associated with AIS. The aim of this study was to examine whether the Scoliosis Research Society-22 (SRS-22) outcome questionnaire reflects the degree of measurable external asymmetry of the back in AIS and thus is a measure of patient outcome for external appearance.

**Methods:**

A total of 102 pre-operative AIS patients were identified retrospectively. Objective parameters were measured using ISIS2 surface topography. The associations between these parameters and the self-image and mental health domains of the SRS-22 questionnaire were investigated using correlation coefficients.

**Results:**

All correlations between the parameters of asymmetry and SRS-22 self-image score were of weak strength. Similarly, all correlations between the parameters of asymmetry and SRS-22 mental health score were of weak strength.

**Conclusion:**

The SRS-22 mental health and self-image domains correlate poorly with external measures of deformity. This demonstrates that the assessment of mental health and self-image by the SRS-22 has little to do with external torso shape. Whilst the SRS-22 assesses the patient as a whole, it provides little information about objective measures of deformity over which a surgeon has control.

## Background

Adolescent idiopathic scoliosis (AIS) is a three-dimensional deformity of the spine typically associated with a range of torso abnormalities including rib and scapula prominences, asymmetry of the shoulders, chest wall deformity and waist asymmetry [1].

Correction of visible deformity is increasingly becoming recognised as an important indication for surgical intervention [2] with one of the goals of surgery being to improve both physical health and health-related quality of life (HRQOL) [3]. Both AIS patients and their parents have associated aesthetic concerns [4, 5], with reduction of visible deformity found to be the second most common reason for patients requesting surgical intervention [5].

In light of the increasing recognition and importance of patient-reported outcomes, attempts have been made to develop objective measures to address patient’s HRQOL. One questionnaire by the Scoliosis Research Society (SRS), the SRS-22 [6], has been validated in pre-operative AIS and adult scoliosis patients and has been shown to have excellent internal consistency and reliability [7–9].

It is established that patients with AIS suffer from reduced HRQOL, often experiencing more pain, impaired function, lower self-esteem and increased rates of depression than their contemporaries [10–13]. A review by Rushton and Grevitt found that, compared to unaffected peers, patients with AIS had statistically worse pain and poorer self-image [14]. Of these SRS domains, self-image was the only one found to be consistently worse clinically.

The traditional measurement for quantifying spinal deformity is the Cobb angle [15], which is a measurement of the size of the curve in the spine in the coronal plane measured on a posterior-anterior radiograph. This measurement assesses spinal deformity in a two-dimensional uni-planar manner. Due to the three-dimensional nature of the deformity in AIS, the use of the Cobb angle has drawbacks and fails to take into account patients’ perceptions of their deformity. Furthermore, several studies have demonstrated that radiological parameters do not correlate well with patients’ subjective perception of body image [1, 16–18]. For this reason, it is increasingly recognised that in addition to radiological measurements, supplementary outcome measures are required to better quantify the deformity [16].

Over the years, new modes of assessing deformity have been developed. Surface topography is one such method allowing a non-invasive, three-dimensional assessment of the surface of the back or torso to be performed, and it has been well validated for assessing spinal deformity in scoliosis [19–23]. Several studies have demonstrated moderate correlation between surface topography and the SRS-22 scores specifically in the self-image and mental health domains [8, 23, 24]. Despite demonstrating these correlations, Brewer et al. [24] concluded that the patients’ view of deformity may be related to other factors that were not fully assessed by their current methodology, highlighting a need to determine additional objective parameters that would better correlate with the patients’ perceptions of their condition.

When attempting to define these additional parameters, reference was made to previous work demonstrating that the shoulder balance, scapula prominence and waistline asymmetry are the most important factors that contribute to overall trunk deformity in AIS patients [25–27].

The purpose of the study was to analyse how well the SRS-22 domains of mental health and self-image reflect the objective parameters of asymmetry measured using the Integrated Shape Imaging System 2 (ISIS2) surface topography system. The overriding aim was to assess whether the SRS-22 questionnaire reflects the measured trunk deformity in areas known to be of concern in AIS and that the surgeon has the opportunity to influence during surgery.

## Methods

This study retrospectively identified 102 pre-operative patients with previously untreated AIS. Patients between 10 and 18 years of age were included. Patients undergoing conservative management with bracing were excluded from the study. The patient cohort was a consecutive series of patients presenting to the spinal clinic at our institution that met the inclusion/exclusion criteria. Each patient had undergone clinical assessment and surface topography using ISIS2 within 6 weeks of completing the SRS-22 questionnaire (mean difference 1 day, SD 6 days, range 0–41 days). Available spinal radiographs were only considered to be appropriate for assessment if taken within 6 weeks of the ISIS2 scan. All patients had undergone a whole spine MRI confirming a diagnosis of idiopathic scoliosis, as is standard practice at our institution. Prior ethical approval was gained (15/EM/0283) through the national ethical approval process.

A perfectly symmetrical back is one without difference between the right and left side of the body. Noting the importance of shoulder balance, scapula prominence and waistline asymmetry [24–26], the following parameters were chosen for use in our study.

**Fig. 1:**
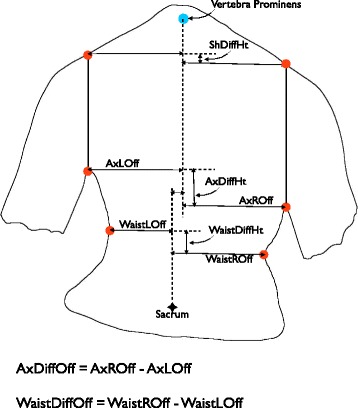
A schematic illustrating asymmetry parameters as measured from a two-dimensional photograph. Reproduced with permission from Gardner et al. [45]

The parameters ‘AxDiffOff’ for the axilla and ‘WaistDiffOff’ for the waist describe the difference (right minus left) in the distances from the midline for points marking the proximal end of the posterior axillary fold and the most medial part of the flank for the waist as shown in Fig. 1. A positive number indicates that the right side had a larger offset than the left. The parameters ‘ShDiffHt’, AxDiffHt and WaistDiffHt describe the difference (right minus left) in the relative heights of the shoulders, axillae and waist in a similar fashion. A positive number indicates that the right side was higher than the left. The parameters AxDiffOff, WaistDiffOff, ShDiffHt, AxDiffHt and WaistDiffHt were all measured from a two-dimensional photograph. The point used for the shoulder in ShDiffHt is marked from a vertical line from the axillary point as that line crosses over the edge of the shoulder girdle.

**Fig. 2:**
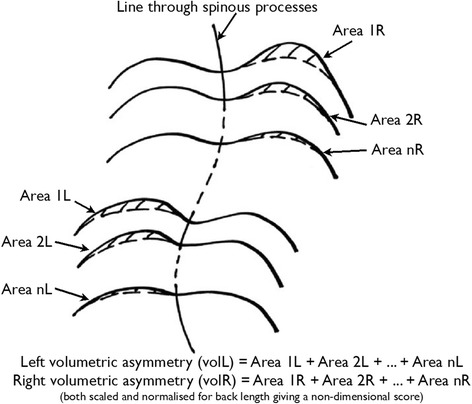
An illustration of how volumetric asymmetry is calculated

The three-dimensional aspect of ISIS2 is defined using volumetric asymmetry. The methodology for this parameter is as follows. Markers are placed on the bony landmarks of the spine and lumbar dimples so that the three-dimensional surface of the back can be related to body axes. A zero plane is defined through the sacrum and the vertebra prominens, parallel to the line running between the markers on the lumbar dimples. A curve is fitted through the markers on the spinous processes on the measured surface and is then used as the axis of symmetry. The difference between the areas of the back surface above the zero plane on each side of the symmetry line is then calculated for each transverse (horizontal) section and allocated to the higher side. The left and right volumetric asymmetry parameters are then calculated by summing the area differences on each side and normalising for back length, as shown in Fig. 2. The parameters ‘VolL’ and ‘VolR’ give objective values for the size (volume) of any rib or lumbar humps seen on the back. A new parameter ‘VolSum’ is defined as the sum of VolL and VolR. ‘VolDiff’ is defined as the difference of VolR minus VolL. These parameters give a measure of the total amount of asymmetry (right and left together) and the difference in the asymmetry between the two sides. An additional parameter ‘ZScapDiff’ is defined as the difference in magnitude between the maximum point (maximum height away from the zero plane) in the left and right scapular areas. These parameters give a measure of the three-dimensional asymmetry of the back.

Modifications were coded adding to the standard ISIS2 user interface to allow the user to locate the positions of the waist creases, axillae and shoulders by identifying these points with the mouse. The remaining parameters based on the standard ISIS2 parameters were calculated automatically as normal [21]. The analysis was carried out by a single researcher (AG) on the new two-dimensional parameters based on the manual identification of the waist, axilla and shoulder locations. The magnitudes of the radiographic spinal curves were measured using the Cobb angle method by the treating surgeon using Picture Archiving and Communication System software (GE Systems, New York, NY, USA).

The relationships between the scores for the SRS-22 self-image and mental health domains and the surface topography parameters were investigated using either the Pearson correlation coefficient (r) or Spearman’s rank correlation coefficient depending on distribution of data type. R software was used for all data analysis [28]. The strength of correlation is defined as 0–0.29 is weak, 0.3–0.69 is moderate and 0.7–1.0 is strong [29]. Statistical significance was set at *p* < 0.05.

## Results

Of the 102 patients included in the study, six (5.9%) were males and 96 (94.1%) females. The mean age of the patients at time of assessment was 14.3 years (standard deviation 1.29 years, range 11.32–17.6 years). Of the 102 patients, only 54 had an appropriate accompanying radiograph. There were 39 patients with Lenke type 1 curves, 13 with Lenke type 3 curves and two with Lenke type 5 curves.

Median total SRS score was 3.30 (interquartile range 2.91–3.82); median self-image score was 2.65 (interquartile range 2.20–3.15) and median mental health score was 3.38 (interquartile range 2.80–4.00). Median Cobb angle was 66.0° (interquartile range 54.0–74.8°).

**Fig. 3:**
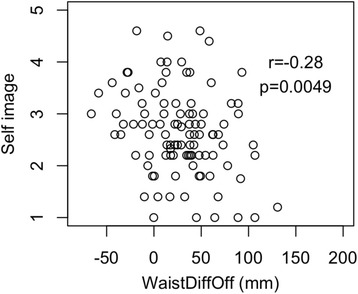
A scatterplot of WaistDiffOff versus Scoliosis Research Society-22 self-image score

Table 1 shows the statistics for the parameters of asymmetry and the SRS-22 questionnaire. All correlations between the parameters of asymmetry and SRS-22 self-image score were of weak strength. Similarly, all correlations between parameters of asymmetry and SRS-22 mental health score were of weak strength. Scatterplots of the SRS-22 self-image and mental health domain scores against parameters of asymmetry were drawn, but none showed a strong relationship. A sample scatterplot for WaistDiffOff and SRS-22 self-image is shown in Fig. 3.

**Table 1:** A table of correlation coefficients and *p* values from parameters of asymmetry compared with Scoliosis Research Society–22 self-image and mental health domains

	Self-image	Mental health
ShDiffHt	*r* = 0.06	*r* = 0.01
	*p* = 0.58	*p* = 0.94
AxDiffHt	*r* = − 0.16	*r* = − 0.21
	*p* = 0.10	*p* = 0.033
WaistDiffHt	*r* = 0.24	*r* = 0.10
	*p* = 0.014	*p* = 0.31
AxDiffOff	*r* = − 0.17	*r* = − 0.23
	*p* = 0.084	*p* = 0.02
WaistDiffOff	*r* = − 0.28	*r* = − 0.22
	*p* < 0.01	*p* = 0.027
VolDiff	*r* = − 0.26	*r* = − 0.13
	*p* < 0.01	*p* = 0.19
VolSum	*r* = − 0.22	*r* = − 0.09
	*p* = 0.024	*p* = 0.30
ZScapDiff	*r* = − 0.21	*r* = − 0.15
	*p* = 0.035	*p* = 0.13

Correlation analysis was also carried out on the Lenke 1 subgroup. The results were similar to the whole group, with all measured correlations being of weak strength. Analysis was not done on the Lenke 3 and 5 subgroups because of the low numbers.

## Discussion

It is well established that patients with untreated AIS tend to suffer a reduced HRQOL often experiencing increased pain, impaired day to day function, lower self-image and self-esteem and increased rates of depression than their contemporaries [10–13]. The need to consider HRQOL when deciding treatment strategy is becoming increasingly recognised among clinicians [2] with one of the main goals of surgery now to improve both physical health and HRQOL.

There has been an increasing use of disease-specific, patient-reported questionnaires such as the SRS-22, the Spinal Appearance Questionnaire (SAQ) [30] and the Trunk Appearance Perception Scale (TAPS) [31], to help clinicians assess a patient’s HRQOL and decide on the most suitable management. Furthermore, questionnaires also allow clinicians to assess the impact of a specific management strategy.

Despite Cobb angle being the traditionally accepted standard for measuring the size of a scoliotic curve [15], Brewer et al. [24] demonstrated that volumetric asymmetry correlated better than the Cobb angle with the self-image and mental health domains of the SRS-22 questionnaire. This is not unexpected. Goldberg et al. in their paper of 2001 stated “it is the rib hump that the patient is unhappy with, not the value of the Cobb angle” [20]. The measurement of volumetric asymmetry enables clinicians to better address patient perceptions of their own deformity and in turn goes some way in understanding the psychological impact the resultant deformity has in AIS [11, 13].

Whilst the Brewer et al. study [24] demonstrated better correlation of the SRS-22 self-image and mental health domains with a volumetric asymmetry parameter from surface topography than with Cobb angle, the correlations were only of a moderate level. The authors concluded that volumetric asymmetry alone, as calculated by surface topography, was insufficient to completely explain a patient’s own perception of self-image and mental health in AIS and that additional objective parameters were needed. This led to the development of the anatomical points for the shoulder, axilla and waist as used in this paper as it has been previously demonstrated that shoulder balance, scapula prominence and waistline asymmetry are the most important factors that contribute to overall trunk deformity in AIS patients [25–27]. Using photographic measures to evaluate waistline asymmetry in patients with idiopathic scoliosis, Matamalas et al. [32] demonstrated significant correlation between anatomic landmarks of waistline asymmetry and Cobb angle. Furthermore, a significant, yet weak, correlation between clinical measures of waistline asymmetry and the patients’ perception of their deformity was demonstrated. Whilst considered a key factor in the perception of trunk deformity in scoliotic patients [25, 27], it has recently been suggested that patients’ perceptions of their shoulder deformity do not correspond with clinical measures of shoulder balance. Using clinical photography, Matamalas et al. [33] demonstrated no correlation between clinical measures of shoulder balance and patients’ perceptions of their deformity in non-operated scoliotic patients, calling into question the value of shoulder balance in the overall assessment of trunk deformity. Interestingly in a normal study population, Akel et al. [34] found that 28% had a shoulder imbalance greater than 10 mm. However, all of these people perceived themselves as having balanced shoulders. These findings suggest that in the absence of other aspects of trunk deformity shoulder balance goes unnoticed. In the scoliotic population it is possible that the presence of other aspects of trunk deformity may negatively impact their perception of their own shoulder balance.

This paper adds to the literature by demonstrating that the assessment of external deformity in AIS is not well performed when using the SRS-22 scores. Despite the extensive number of parameters of asymmetry used, our study was only able to identify weak correlations with the SRS-22 self-image and mental health domains. This demonstrates that the assessment of mental health and self-image by the SRS-22 seems to have little to do with measurable external torso shape. Whilst the SRS-22 assesses the patient as a whole, it provides little information about objective measures of deformity over which a surgeon has control during a scoliosis operation, one aim of which is to change torso shape.

It was interesting to note that WaistDiffHt and ShDiffHt demonstrated a positive correlation with SRS-22 self-image and mental health domains, although only WaistDiffHt with self-image was statistically significant. One would expect that as the difference in relative heights between the shoulder and waist points increases, the self-image and mental health domain scores would decrease, demonstrating a negative correlation. The significant unexpected positive correlation for WaistDiffHt could possibly be explained by the difficulty encountered whilst identifying the waist in some patients with scoliosis. The waist crease on the concave side is often clear while the waist on the convex side is not. The ability of surgeons to reliably determine waist and shoulder asymmetry in scoliotic patients has been shown to be poor [26]. It should be noted that all correlations measured here were of weak strength whether in the positive or negative directions.

The SAQ [30, 35], TAPS [31] and SRS-22 [7–9] have all been validated in AIS, with the SAQ validated for use with surface topography [23]. Despite the robustness of the SRS-22, it has been shown to have weak to moderate correlation with scoliosis magnitude measured using the Cobb angle [36]. Bago et al. demonstrated that this problem could be overcome by adding dimensions from a pictorial scale to improve correlation with scoliosis curve magnitude [37]. Both the SAQ and TAPS are pictorial questionnaires with their designs previously described [30, 31, 35]. Whilst both the SRS-22 and SAQ have been identified as having significant floor and ceiling effects limiting their ability to detect change [38, 39], the TAPS questionnaire offers an alternative and has been shown to have lower floor and ceiling effects [31].

No studies are known to have used surface topography to directly compare which questionnaires correlate better with HRQOL in AIS. Several studies have, however, used Cobb angle to do this [40, 41]. Matamalas et al. compared three questionnaires; SRS-22, SAQ and TAPS in idiopathic scoliosis [41]. The study found that all questionnaires demonstrated good internal consistency and correlation with scoliosis magnitude. SAQ and TAPS demonstrated the strongest correlation with each other (*r* = − 0.8) whilst SRS-22 demonstrated medium strength correlation with SAQ (*r* = − 0.67) and TAPS (*r* = 0.46). This finding suggests that pictorial scales such as the SAQ and TAPS might assess different constructs within body image. Both SAQ and TAPS correlated better with Cobb angle compared to SRS-22 self-image (*r* = 0.61, *r* = 0.62 vs. *r* = − 0.41 respectively). Specifically, in younger age groups, there was a lack of correlation between the SRS-22 and Cobb angle, thus questioning the ability of textual scales to address self-image issues effectively in the young, a finding previously highlighted by Parent et al. [38]. Whilst pictorial scales clearly demonstrated a superior ability to address body image, they also correlated lower with the other HRQOL domains than textual scales. This led the authors to conclude that the concurrent use of both pictorial and textual scales would be best to address patient-reported outcome measures in AIS, a view supported in other reviews [40, 42].

There are several limitations to this study. Firstly, both its retrospective nature and method of patient sample selection have inherent shortcomings in terms of study design. Our cohort was a consecutive series of patients presenting to our institution’s spinal clinic. We acknowledge that obtaining a random sample of patients would have been preferential and would have reduced any associated sampling bias. In our cohort, the ratio of females to males (16:1) is greater than the quoted sex ratio for AIS in the literature, where a ratio of 10:1 for curves greater than 30° is reported [43]. This bias towards a greater number of females may have caused a distortion of the results as females and males may react differently to the perceived aesthetic effects of their scoliosis [44]. Secondly, study patients may well have had concomitant mental health issues that were not necessarily a result of their scoliosis meaning that we may have been measuring the psychological consequences of other unrelated issues.

Future work should look to repeat the methodology described in this study but employing the concurrent use of the SAQ, TAPS and SRS-22 questionnaires to assess which questionnaire best addresses different facets of patient HRQOL in AIS. Future development of a combined pictorial and textual questionnaire to assess outcome measures in AIS should be considered.

## Conclusion

Despite extensive use of surface topography parameters known to be important to patients, only weak correlations to the SRS-22 mental health and self-image domains could be demonstrated. Whilst the SRS-22 assesses the patient as a whole, it provides little information about objective measures of deformity over which a surgeon has control.

## Abbreviations

AIS: Adolescent idiopathic scoliosis
HRQOL: Health-related quality of life
ISIS2: Integrated Shape Imaging System 2
SAQ: Spinal Appearance Questionnaire
SRS: Scoliosis Research Society
SRS-22: Scoliosis Research Society-22
TAPS: Trunk Appearance Perception Scale
